# Structure, Activation, and Regulation of NOX2: At the Crossroad between the Innate Immunity and Oxidative Stress-Mediated Pathologies

**DOI:** 10.3390/antiox12020429

**Published:** 2023-02-09

**Authors:** Cristina Nocella, Alessandra D’Amico, Vittoria Cammisotto, Simona Bartimoccia, Valentina Castellani, Lorenzo Loffredo, Leonardo Marini, Giulia Ferrara, Matteo Testa, Giulio Motta, Beatrice Benazzi, Fabio Zara, Giacomo Frati, Sebastiano Sciarretta, Pasquale Pignatelli, Francesco Violi, Roberto Carnevale, Smile Group

**Affiliations:** 1 Department of Clinical Internal, Anesthesiology and Cardiovascular Sciences, Sapienza University of Rome, 00161 Rome, Italy; 2 Department of Movement, Human and Health Sciences, University of Rome “Foro Italico”, 00135 Rome, Italy; 3 Department of General and Specialistic Surgery “Paride Stefanini”, Sapienza University of Rome, 00161 Rome, Italy; 4 Faculty of Medicine and Surgery, Course E, Sapienza University of Rome, 04100 Latina, Italy; 5 Department of Medical-Surgical Sciences and Biotechnologies, Sapienza University of Rome, 04100 Latina, Italy; 6 IRCCS Neuromed, Località Camerelle, 86077 Pozzilli, Italy

**Keywords:** NOX2, oxidative stress, inflammation, immunity, therapeutics

## Abstract

Nicotinamide adenine dinucleotide phosphate (NADPH) oxidase (NOX) is a multisubunit enzyme complex that participates in the generation of superoxide or hydrogen peroxide (H_2_O_2_) and plays a key role in several biological functions. Among seven known NOX isoforms, NOX2 was the first identified in phagocytes but is also expressed in several other cell types including endothelial cells, platelets, microglia, neurons, and muscle cells. NOX2 has been assigned multiple roles in regulating many aspects of innate and adaptive immunity, and human and mouse models of NOX2 genetic deletion highlighted this key role. On the other side, NOX2 hyperactivation is involved in the pathogenesis of several diseases with different etiologies but all are characterized by an increase in oxidative stress and inflammatory process. From this point of view, the modulation of NOX2 represents an important therapeutic strategy aimed at reducing the damage associated with its hyperactivation. Although pharmacological strategies to selectively modulate NOX2 are implemented thanks to new biotechnologies, this field of research remains to be explored. Therefore, in this review, we analyzed the role of NOX2 at the crossroads between immunity and pathologies mediated by its hyperactivation. We described (1) the mechanisms of activation and regulation, (2) human, mouse, and cellular models studied to understand the role of NOX2 as an enzyme of innate immunity, (3) some of the pathologies associated with its hyperactivation, and (4) the inhibitory strategies, with reference to the most recent discoveries.

## 1. NOX2 Isoform of NADPH Oxidase: Activation and Regulation

### 1.1. NOX2 Structure

The Nicotinamide Adenine Dinucleotide Phosphate (NADPH) Oxidase complex (NOX) is a family of reactive oxygen species (ROS)-producing enzymes, first identified in the membrane of neutrophils, where it participates in non-specific host defense against microbes ingested during phagocytosis. In fact, NADPH oxidase and ROS production play a key role in host defense against microbial pathogens.

The NOX family comprises seven isoforms (NOX1, NOX2, NOX3, NOX4, NOX5, DUOX1, DUOX2) that share structural similarities, but differ according to tissue distribution and regulatory systems.

As recently reviewed by Vermont et al. [1], in the timeline of the major steps that led to the identification and description of the NADPH oxidase family, the first observation about the ability of phagocytes to produce ROS was in 1932 [2] when Baldridge and Gerrard demonstrated overconsumption of oxygen during phagocytosis. Afterward, it has been clearly demonstrated that oxidative phosphorylation is not essential for the maintenance of phagocytic activity in polymorphonuclear leukocytes and that mitochondrial inhibitors did not hinder this respiratory burst as most of the oxygen uptake, especially during phagocytosis, is not cytochrome linked [3]. These observations led to the identification of an “alternative respiration”. 

Clinical studies on Chronic Granulomatous Disease (CGD) have also greatly contributed to further understanding of NOX’s structure and function. First identified in the 1950s in 12-month-old children [4], CGD is a rare (~1:250,000 individuals) primary immunodeficiency. Patients affected by CGD have leukocytes that can perform phagocytosis, but the lack of ROS production due to several mutations in the genes that encode NOX2 or any of the four regulatory subunits, impair their bactericidal function and provoke recurring and life-threatening bacterial and fungal infections early in childhood. 

The first description of the molecular basis of NOX2 was developed only in 1987: the enzyme consists of a multicomponent complex including the transmembrane flavocytochrome b588, as well as cytosolic protein subunits (p47^phox^, p67^phox^, and p40^phox^) and small G-proteins, Rac1 (in monocytes) or Rac2 (in neutrophils). These components are maintained physically dissociated in the absence of microbial infections and this ensures that the enzyme is dormant in resting cells. When cells are stimulated, the cytosolic components migrate instantly to the membrane where they assemble with the flavocytochrome b558 to form the active enzyme, a process that is tightly regulated by protein-protein interactions and by phosphorylation of p47^phox^. Although the term NOX2 specifically indicates the transmembrane catalytic protein gp91^phox^, it is sometimes used to refer to the entire enzymatic complex.

The flavocytochrome b588 is a heterodimeric complex that comprises the NOX2 (gp91^phox^), a highly glycosylated protein that appears as a broad smear on SDS-PAGE and runs with an apparent molecular mass of ~70–90 kDa and a 22-kDa subunit (p22^phox^) [5]. NOX2 is the catalytic domain that operates the transfer of electrons across the membrane, from NADPH to molecular oxygen. Its domain includes six membrane-spanning helices connected by five loops (designated from A to E from the N-terminal extremity to the C-terminal extremity). The A, C, and E loops are extra cytoplasmatic, whereas the B and D loops face the cytosol. Two b-type heme groups, located between helices III and V, are coordinated by four conserved histidine residues [6]. 

On the other hand, the p22^phox^ protein maintains stability and contains the anchoring site for the cytosolic partner p47^phox^. Recent studies provide new insight into the structure of the auxiliary subunit p22^phox^ and how p22^phox^ assembles with NOX2 to form the NOX2-p22^phox^ heterodimer in the resting state. Results showed a canonical 6-trans membrane architecture of NOX2 and four transmembrane helices for p22^phox^ [7,8].

As for the cytosolic domains, NOX2 comprehends three phox proteins. P47^phox^, or *NCF1*, is a protein composed of 390 amino acids and comprises an N-terminal domain that interacts with lipids to stabilize the NOX2 complex, a domain that binds p22^phox^, and a C-terminal proline-rich region with a basic charge.

The p67^phox^ protein contains an N-terminal extremity, which binds Rac proteins, a highly conserved activation domain, and a C-terminal extremity.

Lastly, the p40^phox^ protein contains a domain, which binds lipids, and an interaction site for p67^phox^. To activate the whole complex, some small guanosine 5′-triphosphate (GTP)-binding proteins are required, such as Rac1 and Rac2, which interact with the p67^phox^ protein.

### 1.2. NOX2 Activation and Regulation

**Figure 1:**
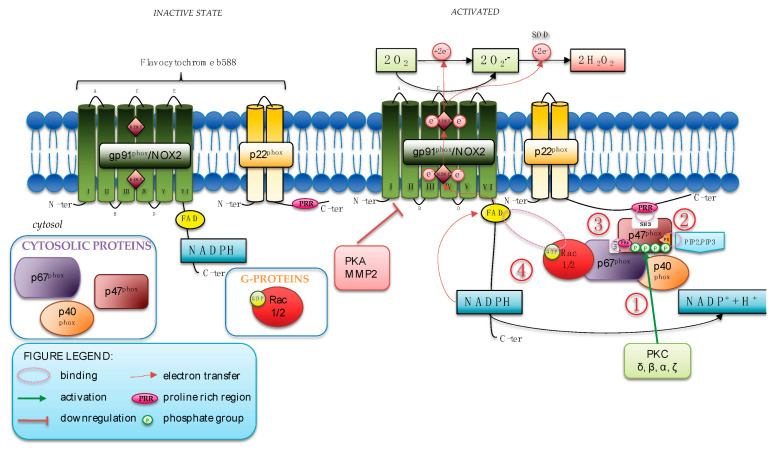
NOX2 activation and regulation. (1) When activated, PKC phosphorylates Ser304, Ser315, Ser320, and Ser328 of p47^phox^; (2) the phosphorylation of p47^phox^ uncovers its N-terminal SH3 domain that then binds the proline-rich region (PRR) in p22^phox^. The PX domain of p47^phox^ binds the products of PI3K; (3) the activation of GTPase Rac2 mediates the translocation of p67^phox^, which associates with p47^phox^ to the cytochrome; (4) Rac2 directly binds to the flavocytochrome favoring the initial steps of the electron transfer reaction.

The sequences of molecular events that occur to activate NOX2 are extensively studied (Figure 1).

During human neutrophil stimulation, assembly of the NOX2 complex requires that p47^phox^ is heavily phosphorylated. The phosphorylation of p47^phox^ uncovers its N-terminal SH3 domain that then binds the proline-rich region in p22^phox^ whereas the PX (phox homology) domain of p47^phox^ binds 3′phosphoinositides, the products of phosphatidylinositol 3-Kinase (PI3K). 

The vital role of this phosphoprotein was first described and identified in CGD patients that demonstrated a selective lack of enhanced phosphorylation in neutrophils after activation of the oxidase with phorbol myristate acetate (PMA) [9]. Several pathways are implicated in p47^phox^ phosphorylation, but a primary pathway involves protein kinase C (PKC) isoforms (δ, β, α, ζ) that, when activated, specifically phosphorylates key serine residues (Ser304, Ser315, Ser320, and Ser328) of p47^phox^. Recently, p67^phox^, alone or in combination with p40^phox^, has been found to be able to potentiate the process of phosphorylation induced by several isoforms of PKC [10]. Thus, p67^phox^ has been suggested as a novel regulator of p47^phox^ phosphorylation, as corroborated by a dramatic reduction in p47^phox^ serine residues phosphorylation in lymphocytes from p67^phox−/−^ CGD patients [10]. 

After this first event of phosphorylation, the activation of the small GTPase Rac2 mediated the translocation of p67^phox^, which associates with p47^phox^ to the cytochrome. Finally, Rac2 directly binds to the flavocytochrome, favoring the initial steps of the electron transfer reaction. 

Data from loss-of-function studies in human primary immunodeficiency and knockout mice highlighted a great deal of mechanistic information on the positive regulation of the NOX2 complex; however, few negative regulators have been identified [11,12,13,14,15]. 

Membrane phosphoinositides have a role in controlling the duration of NOX2 activity [11]. Specifically, Class I PI3K is activated upon neutrophil stimulation and produces Phosphatidylinositol (3,4)-bisphosphate (PI(3,4)P_2_) and Phosphatidylinositol (3,4,5)-trisphosphate (PIP_3_). These products sustain NOX2 activation maintaining the cytosolic subunits at the plasma membrane via the PX domain of p47^phox^ [11]. 

Among negative regulators of NOX2, Noubade et al. identified a new protein, termed negative regulator of ROS (NRROS), which limits ROS generation [12]. Specifically, NRROS directly interacts with nascent NOX2 in the endoplasmic reticulum (ER) and favors NOX2 degradation through the pathway associated with the ER. Indeed, in NRROS-deficient phagocytes, ROS production is increased upon inflammatory challenges; mice lacking NRROS in their phagocytes showed enhanced bactericidal activity but also developed severe experimental autoimmune encephalomyelitis induced by oxidative stress tissue damage in the central nervous system [12]. 

By using genomic technology, several negative regulatory nodes controlling phagocyte oxidative bursts were identified [13]. The authors described mechanisms controlling oxidative burst (1) at the level of NOX2 subunit transcription by the Recombination Signal Binding Protein For Immunoglobulin Kappa J Region (Rbpj) that negatively regulates expression of the NOX2 complex components *NCF1*, *NCF4*, *CYBB,* and *CYBA*; (2) at level of catalytic function by phosphofructokinase, liver type (Pfkl), a rate-limiting glycolytic enzyme that regulates NADPH oxidase activity by affecting the pentose phosphate pathway (PPP) and NADPH levels; (3) at steady-state protein levels (rates of translation and degradation balanced) by Ring Finger Protein 145 (Rnf145) that controls steady-state levels of the NOX2 complex by ubiquitination of lysine residues in gp91^phox^ and p22^phox^ [13].

More recently, cAMP-Protein kinase A (PKA)-NOX2-axis has been proposed as a critical gatekeeper of neutrophil ROS production. Indeed, PKA phosphorylates the cytosolic fragment of NOX2 (291–570) that contains the flavoprotein domain, which exhibits NADPH diaphorase activity. Phosphorylation by PKA induces a decrease in diaphorase activity of the cytosolic protein tail by a conformational change in the NOX2 and downregulates the assembly of the complex by the inhibition of PKC-induced interaction with the cytosolic proteins (Rac2, p67^phox^, and p47^phox^) [14]. 

Finally, our group also described a mechanism of negative NOX2 regulation by metalloproteinases (MMP2), which induces the shedding of sNOX2dp, a small peptide [16,17] that corresponds to the third extracellular domain, the E-loop sequence, of NOX2. As a consequence, NOX2 activity and ultimately ROS formation are downregulated [15]. 

The study of the mechanisms of NOX2 activation and in particular the fine-tuned molecular regulatory systems is of crucial importance since NOX2 can be considered an enzyme at the crossroads between pathologies mediated by NOX2 absence or reduction or NOX2 hyperactivation. 

## 2. The Immune Function of NOX2: Reactive Oxygen Species and Antimicrobial Activity 

NOX2 has an important role in immunity and its structure is clearly linked with its function; as a matter of fact, it is unassembled when inactive. Functionally, the subunit with a considerable role is the gp91^phox^ subunit. Gp91^phox^ is highly glycosylated in the extracytoplasmic portion; the transmembrane portion contains six transmembrane alpha-helices that bind two hemes, whereas in the cytoplasmic portion are located the N-terminal tail and the C-terminal tail which contain NADPH and the binding sites for the FAD [1]. This structure, therefore, contains every characteristic to transport electrons from the cytoplasm to the extracytoplasmic side to produce ROS, which play an important role in killing bacteria. 

Phagocytes are dedicated to microbicidal action thanks to non-oxidants and oxidant systems. Over the years, researchers understood that the two systems cooperate and synergize against bacteria killing. Phagocytes produce different types of ROS that perform this critical function. One of the most important ROS is superoxide ion (O2^•–^), a short-lived molecule with a half-life in the range of microseconds as it is rapidly and normally converted by spontaneous dismutation or enzymatic conversion in H_2_O_2_ [18].

H_2_O_2_ generated by NOX2 must cross phagosome or plasma membranes using aquaporins, integral membrane protein channels that exchange H_2_O_2_ and H_2_O; the molecules rapidly bond with catalases, the main pathways to decompose H_2_O_2_ at higher concentrations [19], whereas peroxiredoxins and glutathione peroxidases are the main routes for H_2_O_2_ metabolism when present at relatively low levels. These enzymes readily react with and manage H_2_O_2_ catabolism and protect critical and vulnerable intracellular proteins from the oxidative stress that could be related to [20]. H_2_O_2_ is often converted into hypochlorous acid (HClO) by the myeloperoxidase (MPO) enzyme, which is abundant in azurophilic granules of neutrophils and in the lysosomes of monocytes. MPO converts H_2_O_2_ to HClO, playing a key role in amplifying the toxicity of H_2_O_2_ generated by the respiratory burst. Indeed, HClO is both a stronger oxidant and antimicrobial agent; in fact, the normal amount generated is adequate to eliminate ingested bacteria. This important role is confirmed by a markedly lesser efficiency at killing bacteria in MPO-deficient neutrophils [21]. 

Neutrophils are usually in a quiescent state and are pre-activated by a process called “priming” that can be established by a range of signaling pathways and intracellular processes causing phenotypic and molecular changes [22]. Even if neutrophils are not able to pre-assemble the NADPH-oxidase complex only with the priming process, they express many receptors on their membrane to facilitate microbial ligands recognition and among them there are toll-like receptors (TLRs), Fc receptors (FcR), G-protein-coupled receptors (GPCR), and integrin receptors that are active in NADPH-oxidase-producing ROS [23]. At the same time as the massive production of oxidants, antimicrobial granule proteins (lactoferrin, myeloperoxidase, defensins, proteases, lysozyme, and calprotectin) are directed to phagosomes or to the extracellular environment to provide for the killing of microbes. 

## 3. Deficit of NOX2: Human, Murine, and Cellular Models 

### 3.1. NOX2 Deficiency: Human Model

NOX2 deficiency, or mutations in one of the genes encoding the components of the NADPH oxidase complex, could lead to the development of chronic granulomatous disease (CGD). CGD is a primary immunodeficiency (incidence 1:200,000–1:500,000) that is due to a defect in the NOX2 complex [24]. According to the mode of inheritance, two classical forms of the CGD are known: an autosomal form, with mutations in genes encoding for p22^phox^ (*CYBA*), p47^phox^ (*NCF1*), p67^phox^ (*NCF2*), and p40^phox^ (*NCF4*) proteins and the X-linked form of CGD with mutations present in *CYBB* encoding NOX2, which accounts for more than 60% of all CGD cases. 

The most common form of autosomal CGD (about 24% of all cases) is caused by mutations in the gene *NCF1* for p47^phox^ where a two-nucleotide GT deletion (ΔGT) occurs as a single defect at the beginning of exon 2 of *NCF1.*

In contrast, a great heterogeneity among mutations was found in X-linked CGD identified in the coding region, introns, and (rarely) in the 5′ flanking regulatory regions of the CYBB gene. The types of mutations included frameshifts (24%), small deletions (11%), nonsense (28.6%) and missense mutations (21.7%), splice-region mutations (19.7%), and regulatory region mutations (0.7%) [25]. 

X-CGD patients with mutations in *CYBB* can be classified into three groups according to the level of cyt*b* 558 expression and NADPH oxidase activity: X91^0^, characterized by an absence of cyt*b* 558 expression and NADPH oxidase activity; X91^−^, characterized by low levels of cyt*b* 558 expression and proportionally decreased NADPH oxidase activity; X91^+^, characterized by normally cyt*b* 558 expression but no NADPH oxidase activity [26]. Recently, 11 new mutations were discovered in 16 CGD male patients classified according to the degree of NOX2 expression and activity. These mutations include a new and extremely rare double missense mutation, a deletion, and a substitution [26]. 

Moreover, a homozygous loss-of-function mutation in *cytochrome b558 chaperone-1* (*Cybc1*) (previously known as *C17orf62*) was identified in patients diagnosed with CGD [27,28]. *Cybc1* encodes for the ER-resident protein EROS (essential for reactive oxygen species), a recently identified protein that acts as a chaperone necessary for the gp91*^phox^*-p22*^phox^* heterodimer expression and controls phagocyte respiratory burst [29]. PLB-985 cells with the deletion of CYBC1/EROS by CRISPR-Cas9 did not express EROS protein and detectable gp91^phox^, whereas p22^phox^ expression was also much lower than in control cells [28]. 

The investigation of X-linked CGD patients has provided a clinical model that can be used to verify the role of the phagocytic NOX2. First manifestations of CGD occur typically during infancy, before the age of 2 years; however, cases of later onset of the symptoms have also been observed in childhood or even adult life [30]. Clinically, CGD is characterized by severe recurrent bacteria, mainly by *Staphylococcus aureus* [31] or *Mycobacterium tuberculosis* [32], and invasive fungal infections [31]. 

In addition to this immunodeficiency, patients suffer from hyperinflammatory reactions. Indeed, NOX2 can serve to resolve inflammation playing a critical role in limiting lung inflammation and injury in response to pathogens, microbial products, and direct tissue injury [33]. Even if key drivers of hyperinflammation are still incompletely defined, some pathomechanisms can include reduced neutrophil apoptosis, dysbalanced innate immune receptors, induction of T helper 17 (Th17) cells, impaired NF-E2–related factor 2 (Nrf2) activity, and increased inflammasome activation [34]. 

However, in association with loss of NOX2 function, CGD patients showed enhanced carotid artery dilation, impaired platelet-related thrombosis, and reduced carotid atherosclerotic burden [35]. In particular, CGD patients have decreased platelet activation as suggested by reduced plasma levels of soluble sCD40 L and soluble P (sP)-selectin, two markers of in vivo platelet activation [36], increased vascular production of nitric oxide (NO) [36] and flow-mediated dilation (FMD) [37], and an index of endothelial function. These data provide a direct link between NOX2, platelet activation, and endothelial function. 

### 3.2. NOX2 Deficiency: Mouse Models

As the deficit of the NOX2 can have important clinical effects, experiments with mouse knockouts lacking NOX2 provide new insights into the mechanisms accounting for CGD development and progression. 

In 1995, Pollock and colleagues generated the first mouse model of X-linked chronic granulomatous disease [38]. In these mice, with a null allele of the gene involved in X-linked CGD, the respiratory burst oxidase activity was completely absent in neutrophils and macrophages; moreover, they manifested typical X-linked CGD phenotypes with increased susceptibility to infection with *S. aureus* and *A. fumigatus* and altered inflammatory response [38]. 

After this first description, genetic deletion is widely used to verify the impact of NOX2-derived reactive ROS [39,40,41,42,43]. More recent reports confirmed that, in mice, the lack of activity of NOX2 in neutrophils, but not in macrophages, markedly increased susceptibility to infectious *A. fumigatus* compared with immunocompetent mice [44]. Moreover, bone marrow-derived macrophages (BMDM) from NOX2-deficient mice failed to clear intracellular *S. aureus,* and showed significantly improved survival and aggravated dissemination of *S. aureus* infection [39]. As above described, besides recurrent bacterial and fungal infections, CGD suffers from hyperinflammation. In a murine model of generalized inflammation induced by zymosan, reproducing systemic inflammatory response syndrome (SIRS), NOX2-deficient mice developed a robust SIRS response, with an enhanced inflammatory phenotype of polymorphonuclear leukocytes and a persistent inflammatory environment resulting from continued chemokine production [40]. Among possible specific drivers of inflammation, leukotriene B4 (LTB4) was found produced at higher amounts by neutrophils from NOX2-deficient mice, also promoting excessive neutrophilic lung inflammation [45]. Moreover, neutrophil-produced interleukin (IL)-1β and downstream granulocyte-colony stimulating factor (G-CSF) were identified as critical amplifying signals that act sequentially with LTB4 to amplify inflammation in CGD mice [46].

Abnormal inflammatory responses are closely associated with many chronic diseases, especially autoimmune diseases. Indeed, effects related to a compromised NOX2 system include the development of autoimmune diseases such as rheumatoid arthritis (RA) [41,42,47], systemic lupus erythematosus [43,48], or psoriasis and psoriasis arthritis [49]. Dark Agouti rats, a model for acute and chronic arthritis, expressed a polymorphic *Ncf1* allele, which led to differences in enzyme activity and expression, resulting in the activation of arthritogenic T cells in lymphoid organs and finally in severe arthritis [41]. NOX2 KO mice spontaneously developed arthritis and the severity was proportionally increased with age. NOX2 deficiency is linked to changes in the development and the modulation of the Th17/Treg immune cell population and promoted inflammatory cytokine production, such as Tumor Necrosis Factor-α (TNF-α), and IL-1β [47]. In a mouse model of pristane-induced lupus, NOX2 deficiency (Ncf1-mutated) aggravated and promoted experimental lupus-like autoimmunity by reducing NETs formation and increasing inflammation [48]. 

### 3.3. NOX2 Deficiency: Cellular Models

Advances in genome sequencing have identified many mutations, opening the door to new and attractive gene therapies that enable a genetic defect to be corrected in the patient’s own cells. However, evaluating these new therapies requires cellular models. The first described in 1993 [50], and the only cell-based model mimicking the X-CGD form, available for this purpose, was the knockout CYBB PLB-985 cell [50,51]. These cells are bi-potential and can differentiate into either granulocytic or monocytic forms. In these cells, the X chromosome-linked gp91^phox^ gene was disrupted by homologous recombination to generate cells that, after differentiation to granulocytes, did not generate O_2_^−^, reproducing the phenotype of the X^0^-CGD patients with this mutation [50]. A second method to generate mutated NOX2 PLB-985 cells was described by Beaumel et al. [51]. PLB-985 cells were transfected with various NOX2 mutations, cultured, and differentiated into neutrophils or monocytes/macrophages to evaluate the impact of these mutations and to study the relationships between NOX2 structure function [51]. 

By using the CRISPR-Cas9 technology, a cellular model of CGD was developed in a monocyte/macrophage THP-1 cell line [52]. The three THP-1 clones generated, harboring different CYBB mutations, displayed phenotypic and functional characteristics of macrophages/phagocytes from CGD patients: reduced CYBB mRNA level, absence of NOX2 expression, and hyper-inflammation state [52]. 

More recently, a method to obtain an ex vivo model of X-CGD has been proposed using induced pluripotent stem cells (iPSCs) [53]. iPSCs, introduced in the year 2006, are artificial stem cells produced from somatic cells and provide a great opportunity as they can be generated from patient cells and differentiated to a desired cell type for disease modeling. X0-CGD cells, reprogrammed from human dermal fibroblasts using episomal vectors, are important to evaluate new therapeutic approaches in preclinical studies. Braut et al. demonstrated the therapeutic potential of NOX2/p22^phox^ liposomes to transport and deliver recombinant cytochrome b558 to the membrane of X^0^-linked CGD (X^0^-CGD) iPSC-derived macrophages and restore the NOX2 activity [54].

## 4. NOX2-Derived Reactive Oxygen Species-Mediated Diseases 

**Figure 2:**
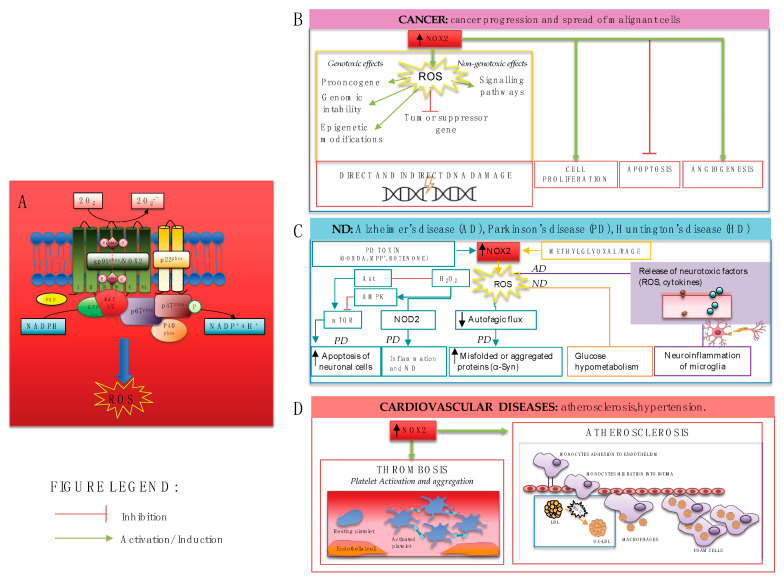
(**A**) NOX2 activation leads to ROS production and contributes to several diseases including (**B**) carcinogenesis, through genotoxic and non-genotoxic ways which may cause direct and indirect damage to DNA, cancer cell proliferation, increased angiogenesis, and inhibition of apoptosis; (**C**) neurodegenerative diseases, through different mechanisms such as apoptosis of neuronal cells in PD via AMPK and Akt/mTOR signaling pathways, the compromising of autophagic flux, neuroinflammation of microglia, and consequent release of neurotoxic factors; (**D**) cardiovascular diseases, such as atherosclerosis and thrombosis through platelet activation, aggregation, and recruitment.

NOX2 has dual functions. As clearly demonstrated by the genetic loss of NOX2 in CGD patients and CGD mouse model, NOX2 is essential for host defense from bacterial and fungal pathogens and calibrates neutrophilic inflammatory response to protect the host from injury associated with excessive or persistent inflammation. On the other side, the hyperactivation of NOX2, as the main source of ROS, is also proinflammatory and injurious, contributing to several diseases, as described in this paragraph (Figure 2).

A compromised antioxidant status and oxidative stress, as characteristic in pathological conditions, such as those described in this paragraph, could be a consequence of a hormonal imbalance [55]. Indeed, changes in the hormonal milieu may have impacts on the production of ROS both directly, as hormones like melatonin, estrogen, or progesterone exhibit antioxidant properties, or indirectly by regulating metabolic activities [55]. Several hormones can influence NOX2. For example, chronic glucocorticoid exposure, secreted by the adrenal cortex, activated NOX2 accelerating neuronal damage in Alzheimer’s transgenic animal models [56]. Additionally, thyroxin can induce NOX2-p47^phox^ expression, finally favoring cardiac hypertrophy in rats [57]. Conversely, melatonin reduced oxidative stress by regulating NOX2 expression in rat lung tissues following whole radiotherapy [58]. 

Oxidative stress and its related diseases could also be influenced by sex hormones [59]. Sex differences have been observed in oxidative stress generation between males and females. Indeed, women seem to be less susceptible to oxidative stress with lower levels of oxidative stress biomarkers and higher antioxidant power [59]. These differences could be explained by the antioxidant effect of estrogen [60,61]. However, at the molecular level, some studies consistently show that NOX2 protein expression did not differ between males and females [59]. 

### 4.1. NOX2 and Carcinogenesis 

In general, ROS formed from NOX enzymes could be a trigger for carcinogenesis in genotoxic and non-genotoxic ways. Genotoxicity refers to direct DNA damage, which may cause proto-oncogene activation, tumor suppressor gene inactivation, genomic instability, and epigenetic modifications, further leading to mutations. Nongenotoxicity describes an indirect effect on DNA through the activation of related signaling pathways [62]. 

Several NOX enzymes are expressed in malignant tissue and may contribute both to cancer progression and the spread of malignant cells. Specifically, NOX2-derived ROS can contribute to carcinogenesis stages, from cell proliferation [63,64] to tumor progression and, finally, to metastasis [63,65,66,67]. 

A recent study analyzed the effect of NOX2 on cell proliferation, cell cycle, cell motility, and cell survival in human esophageal squamous cell carcinoma [63]. Results showed that NOX2 expression favors cell cycle progression; indeed, NOX2 depletion significantly inhibited cell proliferation with the G_0_/G_1_ arrest and resulted in apoptosis. In addition, in osteosarcoma cell lines, high-level NOX2 mRNA expression was observed, and this expression was associated with ROS generation promoting cell survival [64]. When compared with normal tissue, the expression of NOX2 was also significantly increased in primary prostate cancer tissue, in endosomes, promoting cell proliferation and prostate tumor development [66]. The role of NOX2-produced ROS was also confirmed both by the genetic deletion of NOX2 in the mouse model of prostate cancer, resulting in reduced angiogenesis and an almost complete failure in tumor development, and by NOX2 pharmacological inhibition that suppressed established prostate tumors in mice [66].

Studies with genetically NOX2-deficient mice and pharmacologic inhibition of NOX2 also elucidated the role of NOX2 in metastasis [67,68]. Mice deficient in the NOX2 subunit CYBB showed reduced lung metastasis of melanoma cells by downmodulating natural killer (NK)-cell function. The treatment with the NOX2-inhibitor histamine dihydrochloride (HDC) reduced melanoma metastasis and enhanced the infiltration of IFNγ-producing NK cells into the lungs [67]. Moreover, CYBA- or NCF2-deficient mice displayed reduced lung metastatic colonization, the presence of large granulomas of galectin-3 (Mac-2)-positive macrophages and eosinophilic deposits, and altered immune cell populations [68]. 

### 4.2. NOX2 and Neurodegenerative Diseases 

Neurodegenerative diseases (ND) such as Alzheimer’s disease (AD), Parkinson’s disease (PD), and Huntington’s disease (HD), among others, are becoming a wide cause of disability. Growing evidence supports the role of oxidative stress in the initiation and progression of ND [69]. The biochemical integrity of the brain is essential for the central nervous system’s normal function. The brain is highly susceptible to oxidative stress because of its large amount of oxygen consumption and because the neuronal membrane is highly rich in polyunsaturated fatty acids [70]. 

At the cellular level, NOX2 has been reported to be expressed in neurons and astrocytes, [71] and is heavily expressed in microglia, the resident brain phagocytes, where it is involved in immune and inflammatory responses [71,72,73]. Indeed, under normal conditions, microglia serve a crucial role in immune surveillance. However, the overactivation of microglia has been associated with neurodegeneration through the production of neurotoxic factors, such as proinflammatory cytokines and large amounts of ROS [74,75]. 

Alzheimer’s disease, one of the main human dementias in the elderly, manifests as progressive cognitive decline and profound neuronal loss. Senile plaques of amyloid-β (Aβ) peptides and neurofibrillary tangles are the principal neuropathological hallmarks of AD. The role of NOX2 in AD is now well described [76]. In addition, recent studies further supported and reinforced the critical role of NOX2 in the pathogenesis of AD [77,78]. 

In an AD-like model of dementia by the intracerebroventricular (ICV) administration of streptozotocin (STZ), advanced glycation end-products (AGEs), derived from methylglyoxal, activate receptors for AGEs (RAGE) that in turn activate NOX2, that ultimately results in being persistently activated [77]. Methylglyoxal/RAGE/NOX-2 pathway could explain the inflammatory status and oxidative stress in this mouse model and in human disease [77]. Moreover, NOX2-derived ROS-mediated Aβ_1–42_-induced glucose hypometabolism, a symptomatic marker of AD implicated in the initiation of sporadic forms [78]. 

PD is a neurodegenerative disease caused by the loss of dopaminergic neurons and NOX2 plays a critical role in its pathogenesis [79,80,81,82,83,84,85]. First of all, NOX2 is expressed and is activated in dopamine neurons and in microglia both in human brain tissue and animal models of PD [79]. Several mechanisms of the NOX2-mediated effect of oxidative stress in PD have been suggested. NOX2 activation and H_2_O_2_ production are induced by PD toxins, including 6-hydroxydopamine (6-OHDA), 1-Methyl-4-phenylpyridin-1-ium (MPP^+^), and rotenone, favoring AMPK and Akt/mTOR signaling pathways and apoptosis in neuronal cells [80]. NOX2-mediated oxidative stress also induced the expression of nucleotide-binding oligomerization domain-containing protein (NOD)2 and the inflammatory response induced by the neurotoxin 6-OHDA, ultimately promoting DA degeneration [83]. Finally, another mechanism proposed is the impairment of autophagy flux that is implicated in the elimination of misfolded and aggregated proteins such as α-synuclein (α-Syn). Yan et al. found that in a mouse model of PD induced by MPTP, a nigrostriatal dopaminergic neurotoxin, the protein expression of NOX2 increased coincidentally with increased α-Syn Ser129 and reduced autophagy flux, suggesting a role for NOX2-mediated oxidative stress and autophagy in PD pathogenesis [81].

### 4.3. NOX2 and Cardiovascular Diseases 

Oxidative stress has been implicated in the pathogenesis of cardiovascular diseases [86] and NOX2 has emerged as the primary source of ROS in vascular diseases, such as hypercholesterolemia [87,88,89,90,91,92,93] atherosclerosis, and thrombotic complications [35] as well as in cardiac diseases, including myocardial infarction (MI) [94,95,96,97,98,99]. 

The key stages of the atherosclerosis process include the accumulation and oxidation of low-density lipoproteins (LDL) by ROS within the arterial wall and the perpetuation of the inflammatory process occurs through the infiltration of monocyte-macrophages, which become foam cells on the absorption of oxidized LDL. NOX2, by activated platelets, oxidizes LDL, which in turn amplifies platelet activation via ox-LDL receptors CD36 or LOX1 [88]. ox-LDL production was more pronounced in hypercholesterolemic adult patients [88] and also in children with obesity and/or hypercholesterolemia associated with NOX2 activation and reduced flow-mediated dilation [89]. Data from hypercholesterolemic mice models also confirmed the role of NOX2 [91,92]. Apolipoprotein E-deficient (ApoE(−/−)) mice, fed a high-fat diet, showed greater superoxide production by NOX2 and reduced basal nitric oxide-mediated relaxation compared to wild-type mice [92]. Moreover, in hypercholesterolemia mice, NOX2 impairs neovascularization and blood flow recuperation after surgically-induced hindlimb ischemia [91].

Several studies have consistently demonstrated a key role for NOX2 in eliciting platelet activation and aggregation [100,101]. Human platelets express NOX2, which is functionally relevant as indicated by the fact that ROS influence platelet recruitment and thrombus growth [102]. 

Atherosclerosis is a main cause of myocardial infarction (MI), establishing a vicious circle that increases atherosclerosis and the risk of more infarctions [103]. NOX2 is notably upregulated in peripheral blood samples of patients with acute MI [95]. Moreover, in the brain tissue obtained at autopsy from patients with MI, NOX2 is significantly increased in brain microvasculature compared to controls, suggesting also a role for NOX2 in mental health disorders associated with MI [96].

Finally, interestingly, the offspring of patients with early MI had higher NOX2 activation suggesting a key role of NOX2-mediated oxidative stress in the offspring of patients with premature MI [94].

These data support the role of NOX2 as a key source of ROS in the artery wall in conditions that underlie atherogenesis contributing to endothelial dysfunction and vascular inflammation. Therefore, for clinical purposes, NOX2 represents a potential target to develop novel isoform-selective drugs to prevent or treat cardiovascular diseases [104]. 

## 5. NOX2 as a Therapeutic Target: Pharmacological Approaches from Natural to Synthetic Small Molecules 

As described above, NOX2 is implicated in many diseases, where both acute and chronic inflammation plays a key role in terms of pathogenesis. For these reasons, NOX2 could be a target for drug development. Indeed, researchers are looking for inhibitors of NOX2 as a novel therapeutic class of drugs to treat diseases such as AD, PD, cardiovascular diseases, and many other different diseases where oxidative stress and inflammation are key drivers. 

However, as clearly shown by X-CGD patients, side effects could arise from targeting NOX2, including the possibility that such inhibition can contribute to increased infections and/or autoimmune disorders. Therefore, the development of specific and not toxic inhibitors of NOX2 represents to date a still open challenge in the search for the ideal inhibitor. Ideally, this inhibitor should possess specificity and selectivity for the NOX2 isoform, without interfering with its expression or modifying the upstream pathway. Furthermore, the inhibitor should not have antioxidant activities as a ROS scavenger [105].

The first molecules to be investigated as NOX inhibitors and the most frequently used in in vitro and in vivo experiments are diphenyleneiodonium chloride (DPI) and apocynin (4′-hydroxy-3′-methoxyacetophenone/acetovanillone). Apocynin is frequently used as a NOX2 inhibitor even if several studies demonstrated that apocynin acts as an antioxidant [106] and was inactive for NOX2 or any other NOX isoform, even at high concentrations (300 μM) [107]. DPI is an effective low-micromolar or nanomolar inhibitor widely employed in cell studies as a negative control to assess NOX activity; it is a general blocker of flavoproteins, and for this reason, it is not specific for the NOX2. Indeed, DPI is reported to interfere with xanthine oxidase, cytochrome P-450 reductase, and proteins of the mitochondrial electron transport chain [108]. Moreover, the DPI mode of inhibition implies that it will act only on activated NOXs [108]. 

To date, several other small molecules have been identified and tested as NOX2 inhibitors. 

In this paragraph, we reviewed inhibitors tested for NOX2 as a target in pathologies, mainly those described in the previous paragraph (vascular, cancerous, neurological). These inhibitors have been divided into compounds with a direct or indirect mechanism of inhibition. Inhibitors work directly to regulate NOX2 activity by competing for the NADPH binding site of NOX2, or by blocking the assembly of cytosolic subunits to the cytochrome. Indirect inhibitors work as general antioxidants, or by inhibiting the upstream pathway of NOX2 activation (Table 1). 

Moreover, NOX2 inhibitors have been classified as peptide-based inhibitors, drug-like small molecules and drugs, and compounds of natural origin. 

### 5.1. Peptide-Based Inhibitors

Among strategies to target NOX2, peptide-based inhibitors still represent a class of promising candidates because they possess enormous potential and offer specific advantages [154]. By their nature, peptide sequences and peptidomimetics have high similarity to endogenous ligands, high affinity, and low toxicity. Thus, therapeutic peptides effectively and selectively disrupt intrinsic protein–protein interactions [154]. Despite these properties, peptide-based inhibitors showed low stability because of their degradation in the gut and limited oral bioavailability [155]. 

#### 5.1.1. Direct Inhibitor

As a NOX2 inhibitor peptide, the most used is the NOX2ds-tat (NOX2 docking sequence-tat). NOX2ds-tat was, for the first time, designed and described in 2001 [156] as a 9 amino acid sequence from the cytosolic B-loop of NOX2 that interacts with p47^phox^, linked to a 9-amino acid sequence derived from HIV-tat transport region protein, facilitating cellular internalization. NOX2ds-tat is a selective inhibitor of NOX2 as it specifically inhibits O_2_^•−^ production by NOX2 without affecting NOX1- or NOX4-mediated ROS production [157]. 

Bechor et al. identified p67^phox^-derived self-assembled peptides corresponding to an auto-inhibitory intramolecular bond in p67^phox^ located within the 259–279 sequence [109]. In particular, purified peptides 265–270 and 265–279 inhibit NADPH oxidase activity with IC50 = 5.48 µM and 14.99 µM, respectively. Inhibition of oxidase activity by self-assembled p67^phox^ peptides is mediated by binding p67^phox^ protein thus preventing its interaction with NOX2 [109].

#### 5.1.2. Indirect Inhibitor 

New peptide-based inhibitors have been described by Fischer et al., who identified a 9-aa sequence called PLA_2_-inhibitory peptide (PIP) [158]. This peptide derived from the lung surfactant protein A (SP-A) that binds to peroxiredoxin 6 (Prdx6), inhibits its phospholipase A_2_ (PLA_2_) activity, thus preventing the cellular generation of Rac and NOX2 activation [159,160]. PIP-2, the peptide with human sequences, incorporated into liposomes as a delivery vehicle, reduces the phospholipase A_2_ (PLA_2_) activity of peroxiredoxin 6 (Prdx6), called aiPLA_2_, and NOX2 activation in lungs [158]. Moreover, PIP-2 was tested in a mouse model of acute lung injury induced by LPS administration [110]. Pre-treatment of mice with PIP-2 markedly decreased lung injury and mouse mortality [110], suggesting PIP-2 as a useful NOX2 inhibitor to prevent or treat human acute lung injury. 

### 5.2. Drug-Like Small Molecules and Drugs 

As new therapeutic alternatives, small-molecule inhibitors have been successfully used since they offer several key advantages for a targeted approach and are expected to have fewer adverse side effects. Small-molecule inhibitors are compounds with a low molecular weight, less than 500 Da in size [161], that, therefore, can enter whole cell populations easily. They can target any portion of a molecule, both extracellular and intracellular proteins, and can often inactivate their targets rapidly [162]. 

#### 5.2.1. Direct Inhibitor

Among NOX2 small molecule inhibitors, GSK2795039 was the first inhibitor to exhibit promising results in vitro and in vivo [111]. GSK2795039 is a novel 7-azaindole structure with the unique feature of a sulfonamide functionality critical for its activity. GSK2795039 has been reported as a potent NOX2 inhibitor by inhibiting NOX2-mediated activation in cell-free assays (pIC50 6.57 ± 0.17), ROS production in whole-cell assays with human peripheral blood mononucleated cells (PBMCs) (pIC50 6.60 ± 0.075), and in vivo by intravenous infusion in mice [111]. Moreover, GSK2795039 can dose-dependently block NOX2 in models of paw inflammation, and has a protective effect in the mouse model of cerulean-induced pancreatitis [111]. More recently, Xue et al. [112] demonstrated that GSK2795039 may be a potential therapeutic drug for influenza A virus infection. Established the critical role for NOX2 in the H1N1 infection and subsequent inflammatory reactions, GSK2795039 reduced H1N1-induced NOX2 activity and ROS production in human lung epithelial cells coincidentally with decreased expression of pro-inflammatory cytokines such as tumor necrosis factor (TNF)-α, interferon (IFN)-β and interleukin (IL)-6 [112]. 

Several other small molecules were tested as NOX2 inhibitors [113,114,115,116,117,118,119,121]. 

LMH001 is a small chemical compound, recently described as a competitive inhibitor to block phosphorylated p47^phox^ binding to p22^phox^ with an IC50 = 0.149 μM. At a small dose (IC50 = 0.25 μM), LMH001 inhibited angiotensin II (AngII)-induced endothelial NOX2 activation and ROS production by blocking the interaction between phosphorylated p47^phox^ and p22^phox^. Importantly, this small molecule did not have effects on peripheral leucocyte oxidative response to pathogens [113]. In addition, in a mouse model of AngII-induced vascular oxidative stress, hypertension, and aortic aneurysm, LMH001 treatment reduced hypertension, aortic wall inflammation, and incidences of aortic aneurysm, confirming its therapeutic potential [113]. However, a very recent study questions the activity of LMH001 as a NOX2 inhibitor [163]. Indeed, the results of this study showed that LMH001 was chemically unstable in standard aqueous buffer and it was not able to inhibit the p47^phox^/p22^phox^ interaction [163]. 

VAS2870, 3-benzyl-7-(2-benzoxazolyl)thio-1,2,3-triazolo[4,5-d]pyrimidine, is a relatively new compound described in 2006 by ten Freyhaus et al. [164], characterized by NMR and mass spectrometry and identified by NAD(P)H-oxidase specific high-throughput screening [164]. VAS2870 was described as a pan-NADPH oxidase inhibitor, with no relevant specificity for any NOX isoform. Some studies evaluated the effect of VAS2870 on NOX2. These studies demonstrated that VAS2870 by inhibiting NOX2 and ROS production, can restore epithelium barrier integrity in LPS-induced human alveolar epithelial cells [114], and endothelial dysfunction in insulin-treated human adipose microvascular endothelial cell (HAMECs) [115].

Among promising small molecule inhibitors, Phox-I1 is of interest as it specifically binds to the interactive site of p67^phox^ with Rac1 interfering with Rac1-GTP interaction with p67*^phox^* [116]. Platelets treated with Phox-I1 prevented NOX2 activation, ROS generation, and platelet activation in terms of release of P-selectin, secretion of ATP, and platelet aggregation, suggesting Phox-I1 as a possible approach for antithrombotic therapy [117].

Finally, ebselen, previously characterized to have glutathione peroxidase-like catalytic activity, inhibits NOX2 activity in the cell-free assay with an IC50 of 0.6 μM. In PMA-stimulated neutrophils, ebselen, by targeting the proline-rich domain (PRD)-binding site within the *bis*-SH3 domain of p47*^phox^,* completely blocks the association of p47*^phox^* and p22*^phox^* [118,165]. In a mouse model of hyperlipidemia and hyperglycemia (apolipoprotein E/GPx1 (ApoE(−/−)GPx1(−/−))-double knockout (dKO) mice), ebselen significantly reduces NOX2 and oxidative stress ameliorating fibrosis and inflammation in the kidney [119].

As NOX2 inhibitors, drugs with a pleiotropic effect also have a role. Indeed, some drugs normally prescribed for the treatment of several pathologies showed their beneficial effect also through the inhibition of NOX2 [90,122,133].

Statins are hydroxymethylglutaryl-CoA (HMG-CoA) reductase inhibitors used to treat hypercholesterolemia, hyperlipoproteinemia, and hypertriglyceridemia as they lower total cholesterol, low-density lipoprotein (LDL), and triglyceride concentrations while increasing high-density lipoprotein (HDL) concentrations [166].

Among FDA-approved statins, rosuvastatin and atorvastatin showed antioxidant activity by direct NOX2 inhibition [81,90,122,129]. Indeed, rosuvastatin in vitro inhibited platelet NOX2 activation, PKC phosphorylation and p47^phox^ translocation from cytosol to membranes, reducing platelet ROS production and platelet activation [122]. Additionally, in vivo, hypercholesterolemic patients treated with rosuvastatin or atorvastatin showed reduced platelet activation via the inhibition of NOX2-derived oxidative stress [90,122].

#### 5.2.2. Indirect Inhibitors

CYR5099 (4-[(4-(dimethylamino)butoxy)imino]-1-methyl-1H-benzo[f]indol-9(4H)-one) acts as a NOX2 inhibitor as reduced ROS production in stimulated neutrophils, without inhibiting the NOX2 upstream signaling pathways [123]. Moreover, in mice, CYR5099 reduces inflammation, oxidative stress, and edema in complete Freund’s adjuvant (CFA)-induced inflammatory arthritis [123].

BJ-1301 is an aminopyridinol derivative of α-tocopherol with strong antioxidant activity [167]. BJ-1301 is able to block the translocation of cytosolic subunits to the cell membrane, thereby inhibiting NOX2 activation. The subsequent reduction in ROS-mediated receptor tyrosine kinase (RTK) signaling results in the regression of tumor growth in mouse models of lung tumors [124], suggesting BJ-1301-mediated NOX2 inhibition as a promising anti-cancer therapeutics approach.

Dexmedetomidine is a highly selective α-2 adrenergic receptor agonist used in the perioperative period and intensive care units to sedate patients who are under intensive medical care and needs a mechanical ventilator [168]. Dexmedetomidine also exhibits neuroprotective effects in numerous neurological disorders.

By inhibition of hypoxia-induced NOX2 activation in microglia, dexmedetomidine-reduced oxidative stress, [127,128] as indicated by decreased ROS production, malondialdehyde (MDA), and 8-hydroxy-2-deoxyguanosine, as well as increased antioxidant enzymatic activities of SOD and glutathione peroxidase [128], and the neuroinflammatory response [127]. These changes finally alleviated hypoxia-induced cognitive impairment, restored damaged synapses [127], and attenuated apoptosis and neurological deficits [128] in neonatal rats.

Among statins, in the experimental study of coronary micro embolism (CME)-induced cardiac injury, rosuvastatin improved the left ventricular function in these mice, reduced inflammatory cell infiltration and fibrin deposition in the myocardium by inhibiting the expression of NOX2 [129], and also alleviated p53/Bax/Bcl-2-dependent cardiomyocyte apoptosis [129]. Finally, by targeting NOX2, atorvastatin may be identified as a new drug in Parkinson’s disease (PD) treatment [81]. Indeed, atorvastatin treatment improves muscle capacity, anxiety, and depression in MPTP-lesioned mice by inhibiting NOX2 and by promoting autophagy flow [81].

In the treatment of type 2 diabetes (T2D), sodium-glucose co-transporter-2 (SGLT2) inhibitors or gliflozins, a new class of oral anti-diabetic drugs, and liraglutide, a glucagon-like peptide 1 (GLP-1) receptor agonist, are important drugs in glycemic control. Both liraglutide and SGLT2 exert protective effects also by NOX2 inhibition [130,131]. In diabetic rat models induced by streptozotocin (STZ) and a high-fat diet (HFD), liraglutide treatment upregulated the phosphorylation of AMPKα, which prevented NOX2 activation and JNK1/2 phosphorylation alleviating high glucose-induced pancreatic β-cell apoptosis [130]. In T2D patients, gliflozins treatment downregulated NOX2-mediated oxidative stress, thus improving platelet function and thrombus formation [131].

### 5.3. Small Molecules of Natural Origin

The use of natural compounds represents a possible approach for NOX2 inhibition [134,135,136,137,138,139,140,141,142,143,144,145,146,147,148,149,151,152,153]. Among natural molecules, polyphenols represent more than 10,000 compounds occurring naturally in foods such as in tea, chocolate, fruits, and vegetables and are considered highly essential functional foods in a diet with the potential to positively influence human health.

#### 5.3.1. Direct Inhibitors

Among naturally occurring polyphenols, myricitrin is a flavanol that exists in several foods such as tea and different vegetables, and possesses, among others, anti-cancer activities as demonstrated by the induction of pancreatic cancer cell death, attenuation of both DNA strand breakage and mouse skin tumor formation [169]. In a model of acute lung injury induced by LPS inhalation, pre-treatment with myricitrin attenuated the inflammatory process into the airway and alveolar space [135]. Moreover, in the RAW264.7 macrophage cell line, the treatment with myricitrin inhibited the assembly of components of the gp91^phox^ and p47^phox^ thus reducing the intracellular generation of ROS [135].

Ginsenosides are the major, unique, and active components of ginseng and are classified into two categories according to their chemical structure [170]. Among the known ginsenosides, Rb1, and Rg1 have been tested as NOX2 inhibitors.

Rb1 showed specific inhibitory properties by targeting NOX2 for the treatment of atherosclerosis. Indeed, in endothelial cells, Rb1 disrupted the assembly of the NOX2 complex by binding to p47^phox^ and reducing its phosphorylation and membrane translocation. Moreover, in streptozotocin (STZ)-induced ApoE^−/−^ mice, Rb1 reduced aortic atherosclerotic plaque formation and oxidative stress [136].

Celastrol is a natural bioactive ingredient derived from the Chinese herb *Tripterygium wilfordii* Hook. f., an important drug in traditional Chinese medicine with anti-inflammatory and immune-modulating properties [171].

Celastrol potently and effectively inhibited H_2_O_2_ production by several NOXs including NOX2, without inhibiting PKC and the translocation or the binding of p47^phox^ to the stimulated neutrophil membrane [134]. At the molecular levels, celastrol directly binds to p47^phox^ and disrupts the binding of the PRR of p22^phox^ to the tandem SH3 domain of p47^phox^ [134].

Celastrol has been studied for its effects on cardiovascular disorders such as vascular calcification endothelial and endothelial dysfunction [145,146]. Indeed, celastrol is able to alleviate calcific aortic valve disease (CAVD) in a mouse model through the reduction in ROS generation and NOX2-mediated glycogen synthase kinase 3 beta/β-catenin pathway in cultured aortic valvular interstitial cells [146]. Moreover, in endothelial cells, celastrol effectively inhibited the NOX2/Angiotensin II(AngII) type 1 receptor (AT_1_) pathway, improving endothelial cell activity and ameliorating Ang II-mediated HUVEC injury [145].

#### 5.3.2. Indirect Inhibitors

Among polyphenol-rich extracts, wild Alaska bog blueberries (*Vaccinium uliginosum*) were tested as NOX2 inhibitors. At a concentration that exhibited full potency with no apparent cytotoxicity, blueberry fractions disrupted NOX2 assembly by modulating the lipid raft platform and then the association of p67^phox^ to the plasma membrane and abolished TNF-*α*-mediated ROS production [137].

Resveratrol (3,5,4′-trihydroxy-trans-stilbene) is a polyphenol with documented anti-inflammatory, anticarcinogenic, cardioprotective, vasorelaxant, and neuroprotective bioactive effects [172]. In aorta tissue from Wistar rats treated with a high-fat/sucrose diet (HFS) and cultured bovine aortic endothelial cells (BAECs) treated with glucose, resveratrol attenuated senescence of cells and ROS production by inhibition of Sirtuin 1 (SIRT1)/NOX2 pathway [138]. Moreover, in lung epithelial A549 cells, resveratrol attenuated oxidative and inflammatory processes induced by carbon black nanoparticles (CBNPs) by inhibiting NOX2 and p67^phox^ expression [139].

Rosmarinic acid is a natural polyphenol antioxidant found in many Lamiaceae herbs evaluated as a NOX2 inhibitor in the ovalbumin and H_2_O_2_-induced asthma mouse model. The treatment with rosmarinic acid significantly reduced the expression of NOX2, upregulated the activities of antioxidant systems such as SOD, GPx, and catalase, and finally diminished inflammation [140].

Rg1 has been studied for its effects on neurodegenerative diseases [141,142]. Indeed, in a mouse model of AD, amyloid precursor protein/presenilin1 (APP/PS1) AD mice, Rg1 treatment significantly decreased NOX2 expression in the hippocampus and cortex of APP/PS1 mice and then ROS production, finally ameliorating cognitive impairments and neuronal damage [142]. The effect of Rg1 on NOX2 was also confirmed in a mouse model of cerebral ischemia-reperfusion injury, where Rg1 treatment downregulated NOX2 expression and ROS production, attenuating neuroinflammation [141].

Finally, the inhibitory effects of curcumin were also evaluated [149,150,151,152,153]. Curcumin, along with demethoxycurcumin and bisdemethoxycurcumin, is an active component of curcuminoids and exhibits several biological activities, including antidepressant-like actions [149], anticarcinogenic [152], anti-atherosclerotic, and anti-inflammatory activities [151,153].

Curcumin was tested as an antidepressant in chronic unpredictable mild stress (CUMS)-induced depression models in rats [149]. In these rats, curcumin administration relieves depressive-like states by decreasing the protein expression of NOX2 and other biomarkers of oxidative stress such as 4-Hydroxynonenal (4-HNE) and Malondialdehyde (MDA), and increasing the activity of catalase [149].

Curcumin also downregulates NOX2-mediated ROS formation by decreasing the active form of PKC isoforms. Indeed, curcumin can decrease PKCδ reducing p47^phox^ membrane translocation during monocyte-macrophage differentiation, exerting an antiatherogenic effect [151]. Moreover, curcumin, by inhibiting the active form of PKCα, modulated the NOX-2/ROS/ATF-2 pathway, reducing MMP9 expression and lung cancer cell invasion [152].

The described mechanism of action of NOX2 inhibitors and their biological effects highlighted important features that must be taken into account.

Studies on small molecules reported fundamental, pharmacokinetic, or pharmacodynamics profiles highlighting their efficacy. Moreover, most of them work with a direct mechanism of inhibition. Conversely, natural-origin compounds have an indirect effect with limited pharmacokinetic and pharmacodynamic studies. In general, despite the wide distribution and diverse biological properties of naturally occurring compounds, their use is hindered by several limitations. Importantly, these compounds, mainly polyphenols, possess strong functional promiscuity [173]. Promiscuity refers to the ability of confirmed bioactive compounds to specifically interact with multiple targets [174]. Among hundreds of chemical classes, several compounds such as quinones, catechols, and curcumin occur as PAINS (pan-assay interference compound) [175], a term that described an approach introduced by Baell et al. combining expert knowledge of chemical patterns with systematic empirical validation [176]. Therefore, a wider perspective is needed to elucidate whether their use as bioactive components may be valid for the management of diseases.

## 6. Conclusions

NOX2 has a key role in the production of ROS with multiple roles in regulating many aspects of innate and adaptive immunity but also in the pathogenesis of several diseases characterized by increased oxidative stress and inflammatory process.

NOX2 could represent a key target for biological science research. Indeed, researchers are looking for inhibitors of NOX2 as a novel therapeutic class of drugs to treat diseases such as AD, PD, cardiovascular diseases, and many other diseases where oxidative stress and inflammation are key drivers. However, as clearly shown by X-CGD patients, possible side effects could arise from targeting NOX2, including the possibility that such inhibition can contribute to increased infections and/or autoimmune disorders.

The development of suitable molecules to treat these conditions primarily requires a thorough understanding of the regulatory processes of NOX2 activation mechanisms through functional and structural studies. Although numerous inhibitors are tested to date, additional approaches are needed to overcome some issues including (1) the sequence homology between different NOX isoforms which complicates to identify new isoform-selective small-molecule inhibitors; (2) the redox and ROS-scavenging activities of many chemical compounds and (3) the interference with NOX2 upstream pathways.

Therefore, the development of the ideal inhibitor, specific and not toxic, of NOX2 represents, to date, a still open challenge.

## Figures and Tables

**Table 1:** Characteristics, molecular mechanism, and main effects of NOX2 inhibitors.

Compound Name	Pathology	Main Effects	Suggested Mechanism of Inhibition	Type of Study: Range of Concentrations Tested	Ref.
PEPTIDE-BASED INHIBITORS					
DIRECT INHIBITION					
p67^phox^-derived self-assembled peptides	Not applicable	↓ NOX2 activation	p67^phox^ inhibitory peptides	In vitro study: 0.19–50 μM in cell free assay	[109]
INDIRECT INHIBITION					
Peroxiredoxin 6 (Prdx6)-inhibitory peptides	Acute lung injury	↓ ROS production ↓ Phospholipase A_2_ ↓ LPS-mediated lung injury	Inhibition of Prdx6-PLA_2_ activity by the SP-A peptide	Animal study: 2 µg/g	[110]
DRUG-LIKE SMALL MOLECULES AND DRUGS					
DIRECT INHIBITION					
GSK2795039	Inflammation/acute pancreatitis	↓ ROS formation ↓ NOX2 activity ↓ Amylase levels	Competition for the NADPH binding site of NOX2	Animal study: 100 mg/kg	[111]
GSK2795039	Influenza A viruses infection	↓ ROS formation ↓ NOX2 activity ↓ Viral burden	Competition for the NADPH binding site of NOX2	Animal study: 100 mg/kg In vitro study: 0–80 μM in A549 cells	[112]
LMH001	Vascular oxidative stress, hypertension, and aortic aneurysm	↓ AngII-induced ROS production ↓ NOX2 activity ↓ Hypertension ↓ Aortic walls inflammation ↓ Incidences of aortic aneurysm	Blocking p47/p22 binding	Animal study: 2.5 mg/kg In vitro study: 0–100 μM in PBMC	[113]
VAS2870	ARDS	↓ NOX2 expression ↓ ROS production ↑ ZO-1	Covalent ligands of the dehydrogenase domain	In vitro study: 0–20 μM in A549 cells	[114]
VAS2870	Hyperinsulinemia-induced microvascular endothelial cell dysfunction	↓ ROS production ↓ NOX2 expression ↓ p47^phox^ phosphorylation ↑ NO ↑ FID	Covalent ligands of the dehydrogenase domain	In vitro study: 2 μM in arterioles from human skeletal muscle tissue and HAMECs	[115]
Phox-I1	Not applicable	↓ RAC1 binding ↓ NOX2-mediated superoxide production	Binding to p67^phox^	In vitro study: 10 μM in neutrophils	[116]
Phox-I1	Thrombosis	↓ ROS production ↓ P-selectin release ↓ Platelet aggregation ↓ Akt phosphorylation	Binding to p67^phox^	In vitro study: 3–10 μM in platelets	[117]
Ebselen	Not applicable	↓ NOX2 activity	Inhibition of p47^phox^ and p67^phox^ translocation to membranes	In vitro study: 10 µM in human neuthrophils	[118]
Ebselen	Diabetes-associated atherosclerosis/renal injury	↓ NOX2 expression ↓ Oxidative stress ↓ Fibrosis ↓ Inflammation	Inhibition of p47^phox^ translocation to membranes	Animal study: 10 mg/kg	[119]
Tetrahydroisoquinoline analogs (compounds 11 g and 11 h)	Not applicable	↓ NOX2 activity	Disruption of p22^phox^ binding to p47^phox^	In vitro study: 3–300 μM in COS-NOX2 cells	[120]
Perhexiline	Not applicable	↓ NOX2 activity	Unknown	In vitro study: 1 nM–100 μM in human neutrophils	[121]
Rosuvastatin	Hypercholesterolemia	↓ NOX2 activity ↓ Platelet isoprostanes ↓ Platelet recruitment ↓ Platelet PLA_2_	Inhibition of p47^phox^ translocation to membranes	Human study: 20 mg In vitro study: 0.1–10 µM in human platelets	[122]
Atorvastatin	Hypercholesterolemia	↓ NOX2 activity ↓ Platelet isoprostanes ↓ Platelet recruitment ↓ Platelet PLA_2_	Inhibition of p47^phox^ translocation to membranes and Rac1	Human study: 40 mg In vitro study: 1–10 µM in human platelets	[90]
INDIRECT INHIBITION					
CYR5099	Inflammatory arthritis	↓ ROS production ↓ Neutrophil infiltration ↓ Edema	Inhibition of NOX2 upstream pathways.	Animal study: 10–25 mg/kg In vitro study: 1–15 μM in human neutrophils	[123]
BJ-1301	Lung cancer	↓ ROS ↓ NOX2 activity ↓ Cell proliferation Regression of tumor growth	Inhibition of NOX2 upstream pathways.	Animal study: 1–5 mg/kg In vitro study: 0.1–1 μM in endothelial and lung cancer cells	[124]
APX-115	Diabetic nephropathy	↓ NOX2 expression ↓ 8-isoprostane level ↑ Insulin resistance ↓ Mesangial expansion	Attenuation of NOX2 protein expression	Animal study: 60 mg/kg	[125]
GLX481304	Ischemia–reperfusion	↓ ROS production ↑ Contractile function in cells and whole heart	Unknown	In vitro study: 1.57 μM in cardiomyocytes	[126]
Dexmedetomidine	Perinatal Hypoxia	↓ ROS production ↓ NOX2 activity ↓ 4-hydroxynonenal ↓ Proinflammatory cytokines	Reduction in NOX2 expression	Animal study: 25 mg/kg In vitro study: 1 μM in BV2 microglial cells	[127]
Dexmedetomidine	Hypoxic-ischemic brain injury	↓ ROS production ↓ NOX2 activity ↓ MDA ↓ 8-OHdG ↑ Antioxidant activity	Reduction in NOX2 expression	Animal study: 25 mg/kg In vitro study: 1 μM in primary hippocampal neurons	[128]
Rosuvastatin	Coronary Microembolism-induced cardiac injury	↓ ROS production ↓ NOX2 activity ↑ pro-apoptotic proteins ↓ anti-apoptotic Bcl-2	Reduction in NOX2 expression	Animal study: 10 or 20 mg/kg In vitro study: 10 or 20 μM in cardiomyocyte	[129]
Atorvastatin	Parkinson’s disease	↓ NOX2 activity ↓ α-synuclein Ser129 expression ↓ LC3II/I expression ↑ Muscle capacity ↓ Anxiety ↓ Depression	Reduction in NOX2 expression	Animal study: 10 mg/kg	[81]
GLP-1Ra (Liraglutide)	Diabetes mellitus	↓ NOX2 activity ↓ JNK1/2 phosphorylation ↓ AMPKα phosphorylation ↓ β-cell apoptosis	Reduction in NOX2 expression	Animal study: 0.2 mg/kg	[130]
Gliflozins (dapagliflozin)	Type 2 diabetes mellitus	↓ NOX2 activity ↓ ROS production ↓ Platelet activation ↓ Thrombus formation ↑ Antioxidant power	Inhibition of NOX2 upstream pathways	Human study: 10 mg In vitro study: 10 or 20 μM in platelets	[131]
Auranofin	Not applicable	↓ Superoxide anion generation	Inhibition of NOX2 upstream pathways	Human study In vitro study: 0.5–4 μg AU/mL in PBMC	[132]
N-substituted Phenothiazine	Not applicable	↓ NOX2 activity	Unknown	In vitro study: 0.35–50 µM in PLB-985	[133]
COMPOUNDS OF NATURAL ORIGIN					
DIRECT INHIBITION					
Celastrol	Not applicable	↓ H_2_O_2_ production ↓ NOX2 activity	Disruption of the binding of the PRR of p22^phox^ to the tandem SH3 domain of p47^phox^	In vitro study: 0.10–100 μM in human neuthophils	[134]
Myricitrin	Acute lung injury	↓ NO production ↓ TNF-α, IL-6 ↓ Intracellular ROS production	Inhibition of NOX2/p47^phox^ assembly	In vitro: 0–500 μg/mL in RAW264.7 macrophage cells	[135]
Ginsenoside Rb1	Atherosclerosis	↓ p47^phox^ phosphorylation ↓ ROS production	Repression of p47^phox^ activity	Animal study: 50 mg/kg In vitro study: 0–30 μM in endothelial cells	[136]
INDIRECT INHIBITION					
Blueberry-derived polyphenols	Central nervous system	↓ ROS production	Modulation of lipid raft formation and p67^phox^ colocalization	In vitro study: 5 μg/mL in human neuroblastoma cells	[137]
Resveratrol	Senescence of aorta cells induced by HFS	↓ Senescence of aorta cells ↓ ROS production ↓ Expression of p47^phox^ subunit	Downregulation of p47^phox^ protein expression	Animal study: 50 or 100 mg/kg In vitro study: 0.1 or 1 μM in cultured BAECs	[138]
Resveratrol	Inflammation	↓ Expression of NOX2 ↓ ROS production	Downregulation of PKC-α protein expression	In vitro study: 1, 5, and 10 μM in lung epithelial A549 cells	[139]
Rosmarinic acid	OVA-induced lung diseases	↓ IL-4, IL-5, and IL-13 ↓ ROS production ↓ NOX2 expression	Downregulation of mRNA NOX2 expression	Animal study: 10, 20, or 40 mg/kg	[140]
Ginsenoside Rg1	Cerebral ischemia-reperfusion injury	↓ Oxidative stress ↓ NOX2 expression ↓ Calcium overload	Downregulation of NOX2 and NOX2-related protein expression	Animal study: 5, 10 mg/kg In vitro study: 5, 10 μM in HT22 cells	[141]
Ginsenoside Rg1	Alzheimer’s disease	↓ ROS production ↓ NOX2 expression ↓ p-Tau level ↓ APP expression, ↓ Aβ generation	Downregulation of NOX2, p22^phox^, and p47^phox^ mRNA and protein	Animal study: 5, 10 mg/kg	[142]
Higenamine	Neuropathic pain	↓ ROS production ↓ MDA levels ↓ TNF-α, and IL-6 levels ↑ SOD and GSH	Downregulation of NOX2 protein expression	Animal study: 25/50/100 mg/Kg In vitro study: 100/200/400 µM in RSC96	[143]
Dudleya brittonii water extract (DBWE) Polygalatenoside A	Growth of melanoma	↓ Intracellular ROS generation ↓ Mitochondrial activity ↓ ROS generation	Antioxidant effect	In vitro study: 0–90 ng/mL in B16–F10 melanoma cells and NIH/3T3 fibroblasts In vitro study: 0–10 µM in B16–F10 melanoma cells and NIH/3T3 fibroblasts	[144]
Celastrol	Ang II-mediated endothelial dysfunction	↓ ROS generation ↓ NOX2/AT_1_ pathway ↑ endothelial cell activity	Inhibition of NOX2 upstream pathways (ERK1/2/Nrf2)	In vitro study: 50 nM in HUVEC	[145]
Celastrol	Calcific aortic valve disease	↓ ROS generation ↓ Glycogen synthase kinase 3 beta/β-catenin pathway	Downregulation of NOX2 protein expression	Animal study: 1 mg/kg In vitro study: 10 nM in AVICs	[146]
Carnosine	Inflammation	↓ ROS generation ↓ Akt phosphorylation ↓ TNF-α and IL-6 mRNAs ↑ IL-4, IL-10, TGF-β1	Downregulation of NOX2 gene expression	In vitro study: 5–20 mM in RAW 264.7 macrophages	[147]
Ursolic Acid	Liver inflammation and fibrosis	↓ NOX2/NLRP3 signalling pathway ↓ Liver fibrosis	Downregulation of NOX2 gene expression	Animal study: 50 mg/kg	[148]
Curcumin	Depression	↓ NOX2 expression ↓ 4-HNE ↓ MDA	Downregulation of NOX2 protein expression	Animal study: 100 mg/kg	[149]
Curcumin	Seminal vesicle atrophy	↓ NOX2 expression ↓ MDA	Downregulation of NOX2 protein expression	Animal study: 100 mg/kg	[150]
Curcumin	Atherosclerosis	↓ NOX2 expression ↓ p47^phox^ membrane translocation ↓ PKCδ activation	Downregulation of PKC-δ protein expression	In vitro study: 20 µM in monocytes-macrophages	[151]
Curcumin	Lung cancer cells invasiveness	↓ NOX2 expression ↓ p47^phox^ membrane translocation ↓ PKCα activation ↓ MMP9 expression ↓ cell invasiveness	Downregulation of PKC-α protein expression	In vitro study: 0–60 µM in lung cancer A549 cells	[152]
Curcumin	Diabetes-induced vascular inflammation	↓ NOX2 expression ↓ ROS formation ↓ ICAM-1 ↓leukocyte-endothelium interaction	Downregulation of p47^phox^ protein expression	Animal study: 300 mg/Kg	[153]

Legend: 8-OHdG: 8-hydroxy-2′-deoxyguanosine; A549: adenocarcinoma human alveolar basal epithelial cells; AECs: alveolar epithelial cells; APP: amyloid precursor protein; ARDS: acute respiratory distress syndrome; AT1: angiotensin II receptor type 1; AVICs: aortic valvular interstitial cells; BAEC: bovine aortic endothelial cells; GSH: glutathione; HAMECs: human adipose microvascular endothelial cell; HFS: high-fat/sucrose diet; HT22: immortalized mouse hippocampal neuronal cell line; HUVEC: human umbilical vein endothelial cells; LPS: lipopolysaccharide; MDA: malondialdehyde; NLRP3: NLR family pyrin domain containing 3; NO: nitric oxide; OVA: ovalbumin; PBMC: peripheral blood mononuclear cell; Protein kinase C alpha (PKCα); Protein kinase C delta PKCδ; ROS: reactive oxygen species; RSC96: immortal rat Schwann cell 96; SOD: superoxide dismutase; SP-A peptide: surfactant protein A peptide; ZO-1: zonula occludens.

## Data Availability

Data is contained within the article.
